# Iron Content in Deep Gray Matter as a Function of Age Using Quantitative Susceptibility Mapping: A Multicenter Study

**DOI:** 10.3389/fnins.2020.607705

**Published:** 2021-01-06

**Authors:** Yan Li, Sean K. Sethi, Chunyan Zhang, Yanwei Miao, Kiran Kumar Yerramsetty, Vinay Kumar Palutla, Sara Gharabaghi, Chengyan Wang, Naying He, Jingliang Cheng, Fuhua Yan, Ewart Mark Haacke

**Affiliations:** ^1^Department of Radiology, Ruijin Hospital, Shanghai Jiao Tong University School of Medicine, Shanghai, China; ^2^Department of Radiology, Wayne State University, Detroit, MI, United States; ^3^MR Innovations, Inc., Bingham Farms, MI, United States; ^4^SpinTech, Inc., Bingham Farms, MI, United States; ^5^Department of Magnetic Resonance Imaging, The First Affiliated Hospital of Zhengzhou University, Zhengzhou, China; ^6^Department of Radiology, First Affiliated Hospital of Dalian Medical University, Dalian, China; ^7^MR Medical Imaging Innovations India Pvt., Ltd., Madhapur, India; ^8^Human Phenome Institute, Fudan University, Shanghai, China

**Keywords:** magnetic resonance imaging, quantitative susceptibility mapping, age-related brain iron, deep gray matter, multicenter study

## Abstract

**Purpose:**

To evaluate the effect of resolution on iron content using quantitative susceptibility mapping (QSM); to verify the consistency of QSM across field strengths and manufacturers in evaluating the iron content of deep gray matter (DGM) of the human brain using subjects from multiple sites; and to establish a susceptibility baseline as a function of age for each DGM structure using both a global and regional iron analysis.

**Methods:**

Data from 623 healthy adults, ranging from 20 to 90 years old, were collected across 3 sites using gradient echo imaging on one 1.5 Tesla and two 3.0 Tesla MR scanners. Eight subcortical gray matter nuclei were semi-automatically segmented using a full-width half maximum threshold-based analysis of the QSM data. Mean susceptibility, volume and total iron content with age correlations were evaluated for each measured structure for both the whole-region and RII (high iron content regions) analysis. For the purpose of studying the effect of resolution on QSM, a digitized model of the brain was applied.

**Results:**

The mean susceptibilities of the caudate nucleus (CN), globus pallidus (GP) and putamen (PUT) were not significantly affected by changing the slice thickness from 0.5 to 3 mm. But for small structures, the susceptibility was reduced by 10% for 2 mm thick slices. For global analysis, the mean susceptibility correlated positively with age for the CN, PUT, red nucleus (RN), substantia nigra (SN), and dentate nucleus (DN). There was a negative correlation with age in the thalamus (THA). The volumes of most nuclei were negatively correlated with age. Apart from the GP, THA, and pulvinar thalamus (PT), all the other structures showed an increasing total iron content despite the reductions in volume with age. For the RII regional high iron content analysis, mean susceptibility in most of the structures was moderately to strongly correlated with age. Similar to the global analysis, apart from the GP, THA, and PT, all structures showed an increasing total iron content.

**Conclusion:**

A reasonable estimate for age-related iron behavior can be obtained from a large cross site, cross manufacturer set of data when high enough resolutions are used. These estimates can be used for correcting for age related iron changes when studying diseases like Parkinson’s disease, Alzheimer’s disease, and other iron related neurodegenerative diseases.

## Introduction

Iron is ubiquitous in numerous biological processes in normal aging as well as in neurodegeneration. It is distributed throughout the brain in the form of ferritin and its concentration is highest in the deep gray matter (DGM) (Haacke et al., 2005). Iron plays an important role in many brain cellular processes, including oxygen transport, electron transfer, neurotransmitter synthesis, myelination, and mitochondrial function (Drayer, 1986; Connor et al., 1990). Despite this positive role for iron utilization, it is toxic in the form of free iron. In that case, iron can react with oxygen to produce neurotoxic free radicals, which could lead to membrane lipid peroxidation and accumulation of lipofuscin in neurons (Koeppen, 2003). Excessive iron accumulation has been associated with various neuro-degenerative diseases, such as Parkinson’s disease (Ghassaban et al., 2019), atypical parkinsonian disorders (Lee and Lee, 2019), Alzheimer’s disease (Nikseresht et al., 2019), pantothenate kinase-associated neurodegeneration (Álvarez-Córdoba et al., 2019), aceruloplasminemia (Zhou et al., 2020), and different types of hereditary cerebellar ataxias (Deistung et al., 2016). Brain iron deposition is also linked with cognitive severity in Parkinson’s disease (Thomas et al., 2020). For these reasons, probing and quantifying the presence of iron in the brain is very important.

With the common use of 3D multi-echo gradient echo (GRE) imaging methods, the ability to collect whole brain R2^∗^ and quantitative susceptibility mapping (QSM) data has become feasible clinically. QSM is an emerging MRI technique that is sensitive to magnetic susceptibility differences between tissues. The signal phase of GRE sequences can be used to detect the local variations in iron content (Schwarz et al., 2014; Langkammer et al., 2016). R2^∗^ depends on water content as well as iron content and field strength, while QSM is, in principle, independent of water content, echo time, and field strength. QSM has become a complementary method to R2^∗^ for measuring iron content (Liu et al., 2017; Santin et al., 2017). Kofi (2015) assessed the reproducibility of brain QSM in healthy controls (HC) and patients with multiple sclerosis (MS) on both 1.5T and 3T scanners. Brain QSM measurements have good inter-scanner and same-scanner reproducibility for HC and patients, respectively. Ippoliti et al. (2018) also evaluated the reproducibility and consistency of QSM across 1.5T and 3.0T field strengths and optimized the contrast-to-noise ratio (CNR) at 1.5T through bandwidth tuning. Feng et al. (2018) evaluated the repeatability of QSM on a 3.0T scanner using 8 subjects and found that QSM results were highly reproducible across the four time scans. Although the reliability and stability of QSM have been verified in these papers with a small number of cases, a standard from which to calculate the age dependency of iron across manufacturers that also includes the effect of resolution has not been presented or evaluated.

In addition, most current clinical applications of QSM look for differences between patients and HCs in specific brain regions or nuclei (Mostile et al., 2017). Therefore, it is important to be able to correct for age to make a diagnosis relative to patients with a specific neurodegenerative disease. An increase in age-related iron deposition has been reported in many studies. In Hallgren’s landmark work studying brain iron (Hallgren, 1958), histochemical methods were used to show the non-heme iron concentration as a function of age in the brain. Acosta-Cabronero et al. (2016) used QSM to provide insight into iron accumulation in the brain across the adult lifespan (20–79 years old). Whole-brain and ROI analyses confirmed that the propensity of brain cells to accumulate excessive iron as a function of age largely depends on their exact anatomical location. Whereas only patchy signs of iron scavenging were observed in white matter (WM), strong, bilateral, and confluent QSM-age associations were identified in several deep-brain nuclei, chiefly the striatum and midbrain- as well as across motor, premotor, posterior insular, superior prefrontal, and cerebellar cortices. The validity of QSM as a suitable *in vivo* imaging technique with which to monitor iron dysregulation in the human brain was demonstrated by confirming age-related increases in several subcortical nuclei that are known to accumulate iron with age. Their study indicated that, in addition to these structures, there is a predilection for iron accumulation in the frontal lobes, which, when combined with the subcortical findings, suggests that iron accumulation with age predominantly affects brain regions related to motor/cognition/output functions. Keuken et al. (2017) included 30 young, 14 middle-age, and 10 elderly healthy subjects scanned at 7.0T. They investigated volumetric, spatial, and quantitative MRI parameter (T1, T2^∗^, and QSM) changes associated with healthy aging in subcortical nuclei (basal ganglia, red nucleus, and the periaqueductal gray matter). They concluded that aging has a heterogeneous effect across regions. Numerous papers have recently shown a similar relationship with age and iron content using QSM (Aquino et al., 2009; Haacke et al., 2010; Daugherty and Raz, 2013; Hare et al., 2013; Liu et al., 2016; Ghassaban et al., 2018; Yan et al., 2018). However, the number of subjects in these studies was generally small with the largest being 174 (Liu et al., 2016).

Hence, in the present work, our goal was to evaluate the effect of resolution on the QSM quantification; verify the consistency of QSM across field strengths and manufacturers in evaluating the DGM of the human brain using 623 subjects from multiple imaging sites; and establish an iron content baseline (using QSM) as a function of age for each DGM structure using both a global and a regional iron analysis (Liu et al., 2016). Using a normative database of iron content related to age may be useful in categorizing and predicting neurological diseases especially movement and cognition disorder diseases, or conditions which affect motor or cognitive function.

## Materials and Methods

### Data Acquisition

A total of 623 healthy adults were included from 3 sites: The First Affiliated Hospital of Dalian Medical University (Site 1: Dalian), Ruijin Hospital, Shanghai Jiao Tong University School of Medicine (Site 2: Ruijin), and The First Affiliated Hospital of Zhengzhou University (Site 3: Zhengzhou), equipped with a GE HDX 1.5T scanner (173 cases, 85 females, 88 males; age, 45.1 ± 14.2 years; range, 20–69 years), a Philips Ingenia 3.0T scanner (336 cases, 219 females, 117 males; age, 62.3 ± 6.5 years; range, 40–79 years), and a Siemens Prisma 3.0T scanner (114 cases, 61 females, 53 males; age, 60.3 ± 9.3 years; range, 40–90 years), respectively. All the participants provided written informed consent to participate in this study. Data were acquired using the following parameters: TR = 53/25/25 ms, TE = 40/17.5/17.5 ms, and voxel size = 0.6 mm × 0.75 mm × 3 mm = 1.35 mm; 0.67 mm × 1.34 mm × 2 mm = 1.80 mm, and 0.67 mm × 1.34 mm × 2 mm = 1.80 mm for each scanner, respectively.

### Simulation Model

For the purpose of studying the effect of resolution on the QSM data, a digitized model of the brain was used (Buch, 2012). This 3D isotropic model included the general structures of the human brain including the gray/white matter, basal ganglia, and midbrain structures as well as the major veins. The matrix size, voxel resolution and field-of-view (FOV) for this model were: 504 mm × 504 mm × 504 mm, 0.5 mm × 0.5 mm × 0.5 mm, and 252 mm × 252 mm × 252 mm, respectively.

The phase images, $\varphi \left(\vec{r}\right)$, were simulated from the susceptibility model, $\chi \left(\vec{r}\right)$, using the expression (Haacke et al., 2010):

$$\varphi \left(\vec{r}\right)=\gamma B_{0}TEd\left(\vec{r}\right)\text{⊗}\chi \left(\vec{r}\right),\left[1\right]$$

where γ = 2.675×10^8^ *rad*/*s*/*T* is the gyromagnetic ratio and B_0_ = 3T is the main magnetic field strength along the z-direction (the slice select direction in this case); $\vec{r}$ and *TE* are the voxel position vector and the echo time, respectively. Also, ⊗ denotes the convolution operation between $d\left(\vec{r}\right)$, the unit dipole kernel, and the susceptibility model.

**FIGURE 1:**
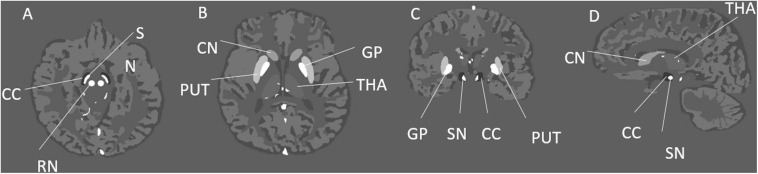
Illustration of the simulated brain model and its structures in three different views. This figure shows different deep gray matter structures such as the: GP, globus pallidus; PUT, putamen; THA, thalamus; CN, caudate nucleus; SN, substantia nigra; RN, red nucleus; and CC, crus cerebri; in the axial **(A,B)**, coronal **(C)**, and sagittal **(D)** views.

Magnitude images were generated from the Ernst equation (Brown, 2014) assuming that *R2*^∗^ $\left(\vec{r}\right)$* = 20/s + 0.125* $\chi \left(\vec{r}\right)$ (Ghassaban et al., 2019). Then, the complex signal was generated from the simulated magnitude and phase images, and Gaussian noise was added to each of the real and imaginary components to produce a signal-to-noise ratio (SNR) of 10:1 at a TE = 7.5 ms. The resulting complex data were then truncated in the slice direction to produce images with the following resolutions: 0.5 mm × 0.5 mm × 0.5 mm, 0.5 mm × 0.5 mm × 1 mm, 0.5 mm × 0.5 mm × 2 mm, and 0.5 mm × 0.5 mm × 3 mm. Figure 1 shows the simulated model in three different views with labels for the main DGM analyzed and Table 1 summarizes the susceptibility values, proton density, T_1_ relaxation times, and the size of the different brain structures in the model.

**TABLE 1:** The susceptibility (ppb), T_1_ relaxation time, relative proton density (ρ_0_), and the size (mm^3^) of different structures for the simulated brain model.

	χ (ppb)	T_1_(ms)	ρ_0_	Size (mm^3^)
WM	**0**	837	0.73	∼
GM	20	1607	0.80	∼
VNT/CSF	−14	4163	1.00	∼
CN	60	1226	0.82	42.5 × 14.5 × 20.5
GP	180	888	0.72	20.5 × 14.5 × 13.5
PUT	90	1140	0.82	33.5 × 14.5 × 21
SN	160, 200	1147	0.79	9.5 × 8.5 × 7, 9.5 × 7.5 × 6
RN	130	833	0.80	6 × 10 × 6
CC	−30	780	0.79	14.5 × 10 × 10.5
THA	10	1218	0.79	29.5 × 26.5 × 17.5
V	450	1932	0.85	∼

*The size is described in row^×^column^×^slice directions format. Please note that SN in the model is composed of two parts. WM, white matter; GM, gray matter; GP, globus pallidus; PUT, putamen; THA, thalamus; CN, caudate nucleus; SN, substantia nigra; RN, red nucleus; CC, crus cerebri; V, veins; VNT, ventricles; CSF, cerebrospinal fluid; and ppb, parts per billion.*

### Resampling of the Data to Create a Single Resolution Common to All Sites

In order to create a comparison with the same resolution, the original data in Site 1 was k-space cropped in-plane to create a resolution of 0.67 mm × 1.34 mm × 3 mm to mimic the in-plane resolution of the other two sites. Likewise, to make the slice thicknesses the same for all sites, the data from the other two sites were k-space cropped through-plane to increase the slice thickness to 3 mm and therefore create an image with the same resolution of 0.67 mm × 1.34 mm × 3 mm.

### Quantitative Analysis

Quantitative susceptibility mapping data were reconstructed using our in-house MATLAB-based toolbox SMART 2.0 (MRI Institute for Biomedical Research, Detroit, MI, United States). The brain extraction tool (BET) (Smith, 2002) was used to isolate the brain tissue (threshold = 0.2, erode = 4, and island = 2000) using the first echo where the signal intensity is highest; a 3D phase unwrapping algorithm (3DSRNCP) (Abdul-Rahman et al., 2007) to unwrap the original phase data; and the sophisticated harmonic artifact reduction (SHARP) (Schweser et al., 2011) to remove unwanted background fields (threshold = 0.05 and deconvolution kernel size = 6). Both BET and SHARP steps were skipped for the simulated data. Finally, a truncated k-space division (TKD) based inverse filtering technique (threshold = 0.1) with an iterative approach (iteration threshold = 0.1, and number of iterations = 4) was used to reconstruct the susceptibility maps (Tang et al., 2013).

**FIGURE 2:**
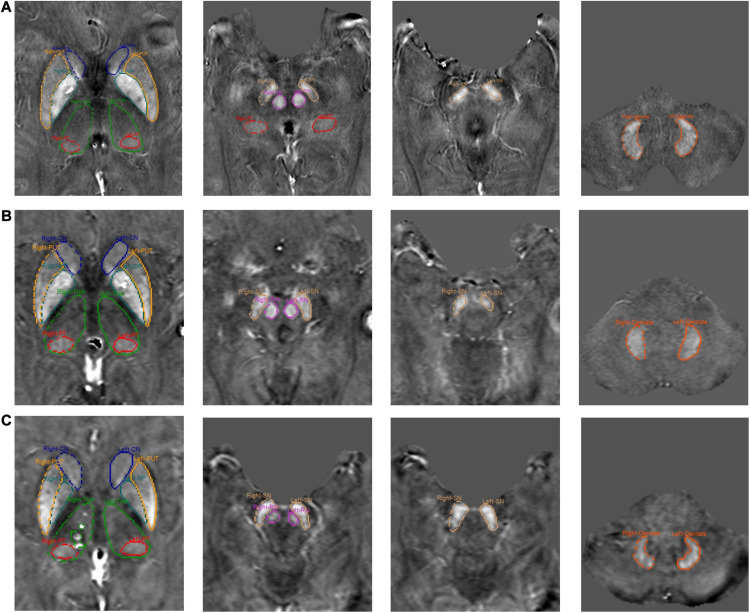
Representative images from each site and segmentation outlines of 8 ROIs. **(A)** Site 1, **(B)** Site 2, and **(C)** Site 3.

Eight subcortical gray matter nuclei, inclusive of caudate nucleus (CN), globus pallidus (GP), putamen (PUT), thalamus (THA), pulvinar thalamus (PT), red nucleus (RN), substantia nigra (SN), and dentate nucleus (DN) were semi-automatically segmented using a full-width half maximum (FWHM) threshold based analysis of the susceptibility maps using Signal Processing in Nmr (SPIN) software (SpinTech, Inc., Bingham Farms, MI, United States). The mean susceptibility values and volumes of the regions of interest (ROIs) were then assessed. Representative images from each site and segmentation outlines are shown in Figure 2. The raters had an intraclass coefficient of >0.9 for the susceptibility measurement of all structures, and these averages are reported in this study. The product of the mean susceptibility and the volume of each GM nuclei was used to represent the total iron deposition in the structure. During the ROI drawing, the readers were blinded to the subject type and age to reduce the impact of ROI selection on evaluating the susceptibility-age relationship. The 3D whole-structural measurements (global) were used to determine age-related thresholds, which were applied to calculate the local iron deposition [RII: portion of the structure that contains high iron concentrations, that is, those regions with iron content higher than two standard deviations above the mean as a function of age as taken from the paper of Liu et al. (2016)]. Age-susceptibility, age-volume and age-total iron correlations were determined for each measured structure for both the whole-region and the high iron content region RII.

### Statistical Analysis

The statistical analyses were performed using MATLAB R2019a (MathWorks, Natick, MA, United States) and SPSS 22.0. Participant demographics were compared between groups with an analysis of variance (ANOVA) or Welch’s ANOVA in the case of nonparametric variables as determined by Levene’s test. Distribution of sex was compared using a χ^2^ test. The mean susceptibility/nuclei volume/total iron content (volume × mean susceptibility) data were fitted using linear regression models (Liu et al., 2016) and Pearson correlation analysis was applied to investigate the relationship between each measure and age in each structure. Strength of the Pearson correlation coefficient (*r*) was determined using the following guide for the absolute value: 0.00–0.19 “very weak,” 0.20–0.39 “weak,” 0.40–0.59 “moderate,” 0.60–0.79 “strong,” and 0.80–1.0 “very strong.” *p*-values less than 0.05 were considered statistically significant.

## Results

### Participant Characteristics

**FIGURE 3:**
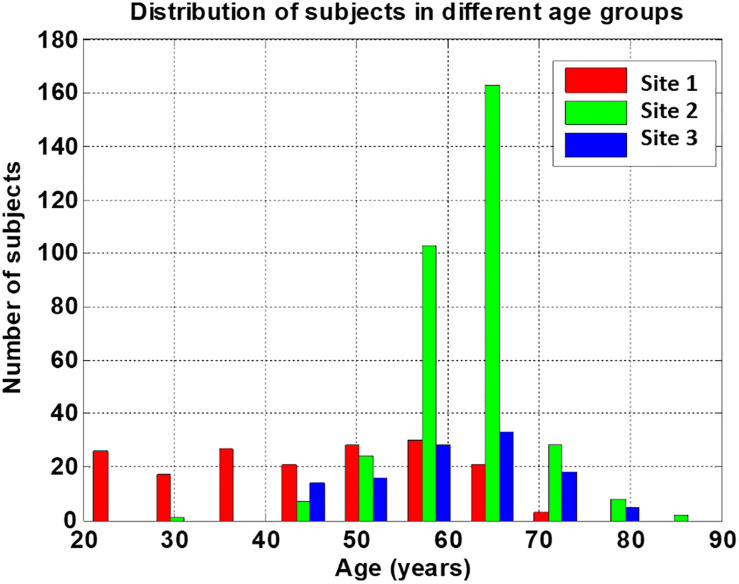
Distribution of subjects’ age from the three sites. Sites 2 and 3 provide needed complimentary data for the higher age range compared to site 1.

Group subject demographics are shown in Table 2. Sex distribution (*χ^2^* = 13.6, *p* < 0.05) and age (Welch’s *F* = 190, *p* < 0.05) differed significantly across the three sites. The average age of the subjects in Site 1 was smaller than that of Site 2 or Site 3, and the distribution of each age group was relatively uniform. The subjects in both sites 2 and 3 were mainly between 55 and 65 years old, as shown in Figure 3.

**TABLE 2:** Demographic data of the subjects and the scanning parameters in three sites.

	Site 1	Site 2	Site 3	*p*-value
Sample size	173	336	114	/
Age range (years)	20–69	40–79	40–90	/
Age (years, mean ± SD)	45.1 ± 14.2	62.3 ± 6.5	60.3 ± 9.3	<0.05
Sex (male/female)	88/85	117/219	53/61	<0.05
MRI scanner	GE HDX	Philips Ingenia	Siemens Prisma	
Field strength (Tesla)	1.5	3.0	3.0	
TR (ms)	53	20	20	
TE (ms)	40	17.5	17.5	
Voxel size (mmł)	0.6 × 0.75 × 3	0.67 × 1.34 × 2	0.67 × 1.34 × 2	

*SD, standard deviation.*

### Effect of Resolution on QSM in the Simulated Data

**FIGURE 4:**
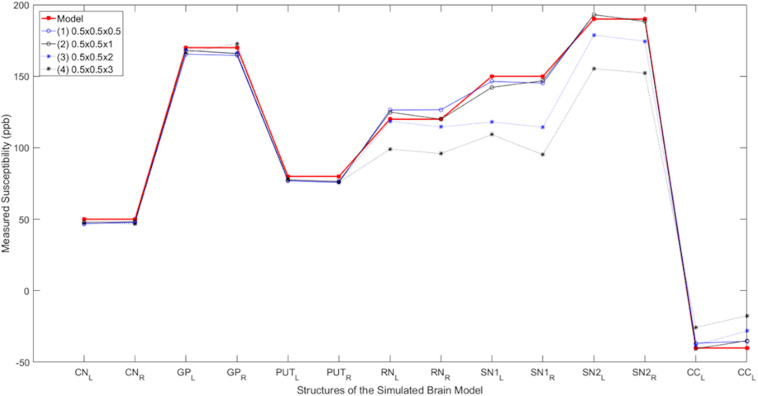
Susceptibility as a function of slice thickness. Comparison of the measured susceptibility values (ppb) of different structures for both left (L) and right (R) sides in the reconstructed QSM data from the simulated noisy phase with different resolutions. The plot in red is the true susceptibility in the original high-resolution model and the other plots show the reconstructed QSM data with different resolutions. As seen in this figure, as the resolution reduces, the measured susceptibility values in smaller structures such as the RN, SN, and CC are negatively affected.

Figure 4 shows the plots of the measured susceptibility values in the various structures of interest in the simulated model for different resolutions. The measured susceptibility values in these plots are zero-referenced with respect to the measured susceptibility in the THA. As seen in these plots, the mean susceptibilities of CN, GP, and PUT are not significantly affected by changing the slice thickness from 0.5 to 3 mm, but for the small structures, such as the SN and RN, the susceptibility is reduced about 10% for 2 mm thick slices and nearly 25% for 3 mm thick slices.

**FIGURE 5:**
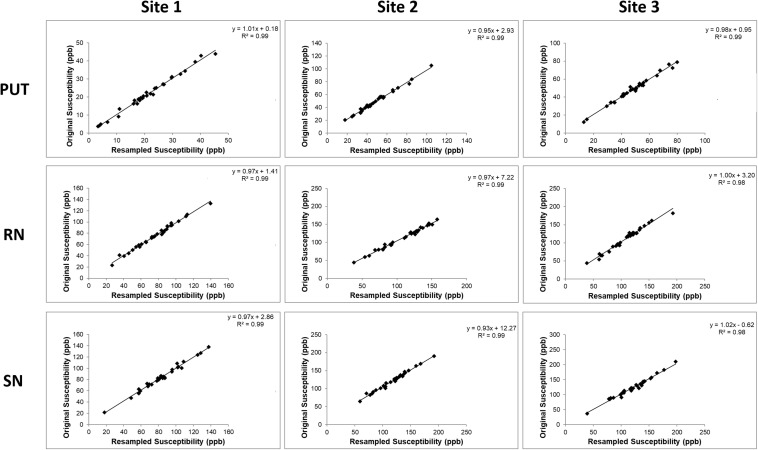
Comparisons between the iron content of the PUT, RN, and SN in the k-space cropped lower resolution images and the original images. These correlations are very high because the same ROIs were used in the cropped and re-interpolated data. When the boundaries are redrawn on the lower resolution data (see Supplementary Figure S5), the correlations are not as good as those shown here, but the data still lies within the 95% confidence intervals as noted in the results section.

The iron content for the k-space cropped lower resolutions were compared to the original iron content measurements (Figure 5). Three representative structures were chosen to highlight in the Figure: the putamen because it is a large structure and the red nucleus and substantia nigra because they are small structures where we expect to see the biggest effect. We found that the *R*^2^ values of the correlations were very high, on the order of 0.9 or higher indicating the closeness of the measurements. The values of all the slopes and correlation measures are given in Supplementary Table 1. For those data that were originally cropped to lower resolution and retraced, the R values were in the range of 0.7 to 0.9 for site 1, 0.8 to 0.9 for site 2 and 0.7 to 0.9 for site 3 for most structures. However, despite not having as high R values as the original data, all the data points in the remeasured lower resolution still fell within the 95% confidence intervals determined by the higher resolution data.

### Relationship Between Mean Susceptibility and Age

#### Global Analysis

**FIGURE 6:**
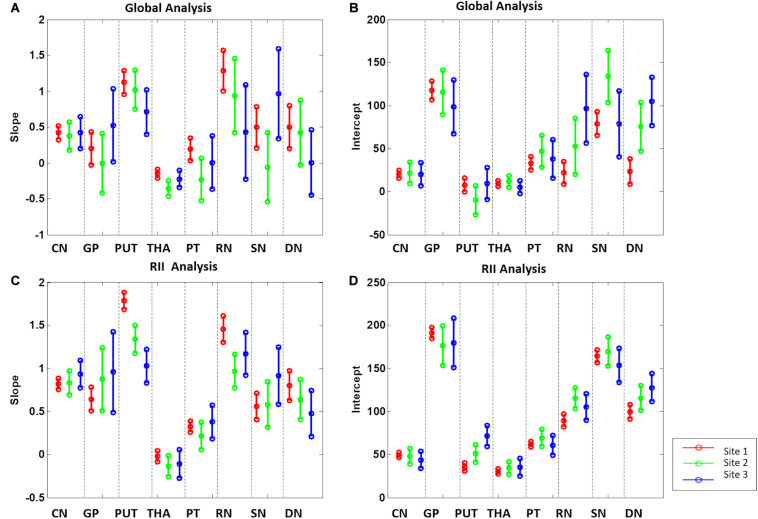
Slopes **(A,C)** and intercepts **(B,D)** 95% confidence intervals of the 8 gray matter nuclei from the 3 sites for the global and RII analysis. CN, caudate nucleus; GP, globus pallidus; PUT, putamen; THA, thalamus; PT, pulvinar thalamus; RN, red nucleus; SN, substantia nigra; DN, dentate nucleus.

**FIGURE 7:**
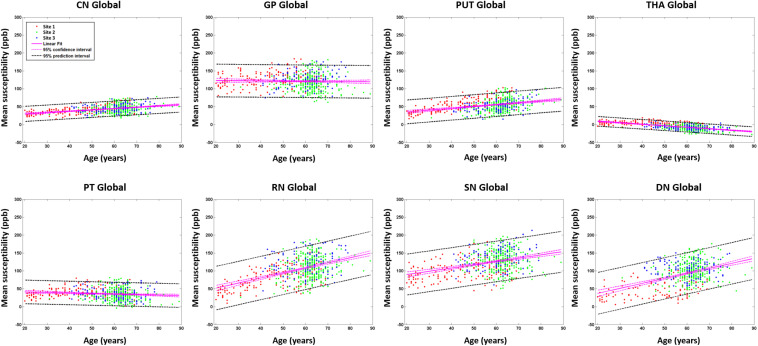
Mean susceptibilities for the global analysis. The mean susceptibility and 95% confidence intervals and 95% prediction intervals are shown for each structure as a function of age. CN, caudate nucleus; GP, globus pallidus; PUT, putamen; THA, thalamus; PT, pulvinar thalamus; RN, red nucleus; SN, substantia nigra; DN, dentate nucleus.

For the global mean susceptibility analysis of iron content, the slopes (ppb/year) and intercepts (ppb) across the sites overlapped for all structures as shown in Figures 6A,B. Further, as shown in Supplementary Table 2, the mean values in the dominant age range (55–65 years) of sites 02 and 03 agree very well with those from site 1 suggesting that the 95% CI projections of site 1 to higher ages should match sites 2 and 3 well. With these two facts in mind, we have merged the data from all 3 sites into a single large dataset to assess the relationship between the mean magnetic susceptibility and age. As shown in Supplementary Table 3 and Figure 7, the mean susceptibility correlated with age (with a positive slope) for the CN, PUT, RN, SN, and DN, all *p* < 0.001. For the THA, there was a strong negative correlation with age (*p* < 0.001). The mean susceptibility in the GP had a negative slope but it was not significantly different from zero (*p* = 0.49) while the PT had a small negative slope that was significant (*p* < 0.01).

#### RII Analysis

**FIGURE 8:**
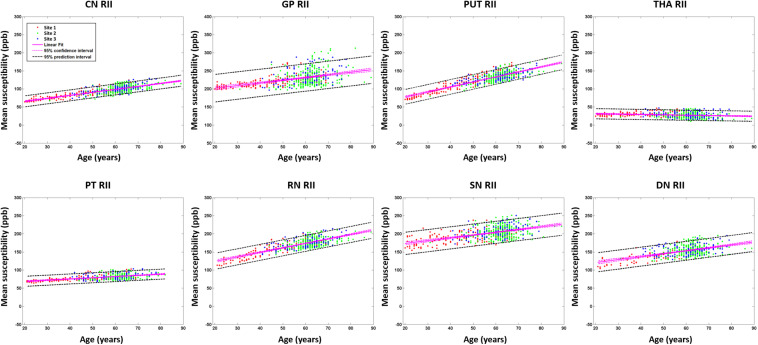
Mean susceptibilities for the RII analysis. The mean susceptibility and 95% confidence intervals and 95% prediction intervals are shown for each structure as a function of age. CN, caudate nucleus; GP, globus pallidus; PUT, putamen; THA, thalamus; PT, pulvinar thalamus; RN, red nucleus; SN, substantia nigra; DN, dentate nucleus.

For the RII regional high iron content analysis, the slopes ranges (ppb/year) and intercepts (ppb) across the sites also overlapped for all structures (except for the PUT and RN) and are shown in Figures 6C,D. Further, as shown in Supplementary Table 2, the mean values in the dominant age range (55–65 years) of sites 2 and 3 agree very well with those from site 1 suggesting that the 95% CI projections of site 1 to higher ages should match sites 2 and 3 well. This allows for the data from the 3 sites to be merged into a single dataset to assess the relationship between the mean magnetic susceptibility and age for RII. For the RII analysis, as shown in Table 3 and Figure 8, mean susceptibility in all the structures was moderately to strongly correlated with age (all *p* < 0.001) except for the THA. For the THA, there was a slight negative slope with age (*p* < 0.001).

**TABLE 3:** Linear fitting equations for mean susceptibility (χ) (ppb) versus age for the RII analysis.

	χ = A × age+B	Error in A (ppb/year)	Error in B (ppb)	*r*	*r*-CI	*p*-value
CN	χ = 0.82 × age+49	0.05	2.89	0.80	(0.77, 0.83)	<0.001
GP	χ = 0.74 × age+187	0.13	7.62	0.43	(0.36, 0.5)	<0.001
PUT	χ = 1.38 × age+50	0.07	3.88	0.86	(0.83, 0.88)	<0.001
THA	χ = −0.1 × age+33	0.05	2.70	−0.17	(−0.25, −0.09)	<0.001
PT	χ = 0.33 × age+62	0.05	2.87	0.52	(0.45, 0.58)	<0.001
RN	χ = 1.23 × age+100	0.09	5.14	0.77	(0.74, 0.81)	<0.001
SN	χ = 0.77 × age+158	0.11	6.18	0.51	(0.45, 0.57)	<0.001
DN	χ = 0.81 × age+105	0.12	6.98	0.53	(0.46, 0.59)	<0.001

*R, Pearson correlation coefficient; r-CI, confidence interval of r.*

### Relationship Between Nuclei Volume and Age

#### Global Analysis

The parameters of the linear fitting equations for the global nuclei volumes versus age are given in Supplementary Table 4. The volumes of most nuclei, including the CN, GP, PUT, THA, PT, and RN were negatively correlated with age (all *p* < 0.001). Of interest is the fact that volumes for the SN and DN both increase with age. Volumes for the global analysis are shown in Supplementary Figure 1.

#### RII Analysis

There was a negative correlation between the volume of the RII region for the THA with age (*p* < 0.001) as shown in Supplementary Table 5. RII volumes of SN and DN increased with age significantly, but correlation between RII volume of RN and age was not significantly different from zero (*p* = 0.14). All the volumes for the RII analysis are shown in Supplementary Figure 2.

### Relationship Between Total Iron Content (Volume × Mean Susceptibility) and Age

#### Global Analysis

The parameters of the linear fitting equations for total iron content globally versus age is given in Supplementary Table 6 and Supplementary Figure 3. Apart from the GP, THA, and PT all the other structures showed an increasing total iron content despite the reductions in volume with age.

#### RII Analysis

The parameters of the linear fitting equations for RII analysis total iron versus age is given in Supplementary Table 7 and Supplementary Figure 4. Like the global analysis, apart from the GP, THA, and PT all structures showed an increasing total iron content (despite the reductions in RII volume with age) as well as a much tighter distribution than the global iron dependence with age.

## Discussion

In this study, the effect of image resolution on the QSM quantification was evaluated based on a simulation model, as well as resampled original data. The consistency of QSM across field strengths and manufacturers in evaluating the DGM of the human brain was verified using a total of 623 subjects from multiple imaging sites, and a QSM baseline as a function of age for each DGM structure for both global and regional iron analysis was determined. The RII analysis showed a much tighter correlation with age and a higher slope than the global analysis for all structures, providing an additional source of information outside whole structure mean iron or total iron for a reliable and sensitive reference for age-related changes. The age related data generated for each structure including susceptibility, volume, or total iron content for both the global and regional analysis can be used to correct for age dependence of each of these measures for monitoring abnormal global and regional iron deposition. The literature and data for RII is scant as it is a relatively new technique for measuring iron on QSM (Habib et al., 2012; Ghassaban et al., 2019; Sethi et al., 2019).

As for the RII analysis findings, the correlations of iron with age are moderate to strong in almost all structures other than the THA, which is consistent with the work of Liu et al. (2016). Even in the GP, which usually shows no iron content change over the lifespan after the age of 20 years (Hallgren, 1958; Xu et al., 2008; Li et al., 2014), we found the Pearson correlation coefficient for age and RII susceptibility greater than 0.40. Data from all major DGM nuclei fell well within the calculated 95% confidence intervals derived from the merged data except for some outliers in the GP. These are likely due to high levels of mineralization in the caudal part of the structure. An added advantage to the RII analysis is that it is less dependent on an accurate drawing of the structure since much of the higher iron content regions are inside the boundaries of these structures. Finally, the slopes and intercepts found by merging the RII data from all three sites agrees with the iron trends with age in other studies (Liu et al., 2010, 2016; Ghassaban et al., 2019).

### Iron Changes With Age

When considering the global analysis for all structures, susceptibility values for the subjects aged 55–65 years are accordance with values recorded by Acosta-Cabronero et al. (2016) on subjects aged 59–79 years. They delineated ROIs of the deep gray matter automatically (CN, PUT, and GP) and manually (RN, SN, and DN) on QSM. When comparing the regression slope and intercepts, there were differences between our study and theirs. One reason may be due to a 3D erosion function being performed to remove spurious pixels whereas our study used a FWHM boundary for our global analysis. But the more likely reason is too narrow an age range without subjects in the 20 to 30 year decade to anchor the data properly. Their team did not report volumetric measurements. The susceptibility versus age plot for the thalamus was flat and similar to our plot, but with a shift in the intercept. Several of the structures have iron content in agreement with recent work by Zhang et al. (2018), who collected data across the human lifespan. The age-related increases in iron affect the morphology and contrast of the structure with the surrounding tissue which is particularly true for the DN. This may have applications to iron rich structures like the SN and GP. Further, we report similar global susceptibility changes with age to a recent work by Ghassaban et al. (2019), who recently used 0.86 mm × 0.86 mm × 1 mm resolution at 3T.

Hallgren and Sourander (Hallgren, 1958) stated that in younger and middle-aged brains, advanced age was associated with greater iron concentrations in the basal ganglia than in subcortical WM, but noted that the association between age and iron content was attenuated after middle age. Much of the recent MR data actually show that there is a continued slow increase in age and the quantity and quality of the published data now by far supersedes the limited results in Hallgren and Sourander.

Of note in these results are the following observations. At any given age, there is a wide range of “normal” iron content. This makes it very hard to give a narrow range of age if one were to be given the iron content. This is less of a problem for the RII analysis because the results are more tightly bound. One might ask what causes this large variation of iron content and if it is related to some disease state like hypertension or vascular disease. This, in itself, would be an intriguing finding to ascertain why some elderly people have the same low iron content as younger people. Furthermore, there is strong neuropathologic evidence that iron overload is a hallmark of many neurodegenerative processes. Outcomes relating to progression of cognitive impairment are of particular interest (Belaidi and Bush, 2016; Moon et al., 2016; Lane et al., 2018; Chang, 2019). Many central nervous system disorders have also been associated with an excessive deposition of iron in specific brain locales as reported in Huntington’s disease (Chen et al., 2013) (caudate, putamen), Parkinson’s disease and multisystem atrophy (Lee et al., 2013; Péran et al., 2018; Seki et al., 2019) (putamen, globus pallidus), multiple sclerosis (Haacke et al., 2009; Mahad et al., 2015; Ropele et al., 2017; Zivadinov et al., 2018) (associated with the MS plaques), and intracerebral hematoma periphery (Garton et al., 2016). An understanding of the normal brain-iron distribution may also help interpret the pathophysiology of the brain damage that occurs in association with neurodegenerative, demyelinating, and vascular disorders.

Another interesting finding is the negative slope of the thalamus. To understand this effect, one has to look back at what QSM actually provides; it provides only changes in susceptibility. The positive and negative values represent more or less magnetic susceptibility relative to the reference region, respectively (Doring et al., 2016). So, if the WM remains the zero mark of QSM then the negative slope of the THA really represents an increasing iron content of the WM with age. Now WM is diamagnetic relative to GM because it is myelinated. However, it has been shown that demyelinated WM has effectively the same susceptibility as GM (about 50 ppb) which makes sense because its overall iron content is about the same as that of gray matter (Haacke et al., 2005; Langkammer et al., 2012). Therefore, an increasing susceptibility of WM with age could represent a general demyelination over time. Since the THA susceptibility changes from roughly 10 ppb to −20 ppb over the age range of 20 years to 90 years, this suggests an increase of WM susceptibility of 30 ppb or a little over half of the difference between healthy WM and GM.

Another consideration is determining the total iron content from susceptibility by estimating the iron concentration from the age equations provided in Hallgren and Sourander’s work as done in other studies (Aquino et al., 2009; Liu et al., 2016). On the assumption that the gray matter density (GMD) remains constant with age, the total iron content is proportional to gray matter volume (GMV). In fact, GMD changes with age, sex, and is not easily quantified (Gennatas et al., 2017). There are a variety of methods that can be used in the future to determine absolute water content and thereby solving this problem; one such method is STrategically Acquired Gradient Echo (STAGE) imaging (Haacke et al., 2020).

### Volume Changes With Age

Our volumetric results for the subjects in the 55-year age range and up are in accordance with recent work by He et al. (2017). Also, Ghassaban et al. (2019) showed volumes decreased with age for all structures (except for SN and DN), suggesting brain atrophy occurs with age. Fjell et al. (2013) measured segmented brain volumes over time for adults 18–94 years of age; while they fit the volumes with an exponential function for age, they noted corresponding decreases in GM volume with age. In another study assessing volumes at 1.5T, Raz et al. (2003) also noted decreases in GM volume in the CN and PUT in a cohort of 55 subjects aged 20–77 years, but not the GP which is highly subject to perivascular spaces and mineralization. Areas of mineralization and large veins were not measured in our ROIs, however, perivascular spaces are not as easily visualized on magnitude on which our tracings were performed and may contribute to the discrepancy in the age versus volume plots between these studies.

For SN and DN, there is a small positive correlation with age, possibly because the increased iron content makes the structures clearer on the QSM data. Our volumetric results, nevertheless, are in accordance with a work which involved 38 subjects, aged 64.1 ± 7.5 years, with DN volumes ranging between 600 and 800 mm^3^ (He et al., 2017). For the SN, the volumes for our analysis overlap well with data for 20 controls (mean age, 60.8 ± 8.3 years, SN volume = 400–650 mm^3^) (Pyatigorskaya et al., 2018). Likewise, total iron content followed the same trends.

### Resolution Effects

As far as resolution is concerned, the large structures are not much affected by slices as thick as 3 mm but the SN, RN and DN are expected to have lower susceptibilities for slices that are too thick. The final effects of these thicker slices also depend to some degree on how the structures are drawn and if partial volume effects are considered. The advantage of tracing the DN on QSM, instead of T2W or SWI, is that the variability of the structural volume is reduced (He et al., 2017). Our group opted to use FWHM to trace the structures which may confer a high reliability between the raters, however, for structures which have smaller size and more intricate detail like the DN, it may be a drawback. Ideally, an automated means to assess the structures may improve the agreement between all structures, field strengths and manufacturers. Both our simulations and the restructuring of the data show that even with resolutions as low as 0.67 mm × 1.34 mm × 3 mm, iron content with age still follows the pattern as shown in this paper, although some minor deviations can occur for smaller objects such as the RN and SN. Nevertheless, for sufficient SNR, higher resolution is always desirable for better edge definition and volume measurements (such as the higher resolution used for sites 2 and 3: 0.67 × 1.34 × 2 mm^3^).

### Limitations

There are several limitations to this study. First, the distribution of subject age was different between site 1 and the other two sites as was the slice thickness. Nevertheless, for larger structures this did not affect the susceptibilities significantly. Second, other QSM methods may provide slightly higher absolute levels of susceptibility by 5 to 10% but, as long as any one method is used consistently across sites and imaging parameters, then the results should be consistent with those presented herein (Liu et al., 2016).

## Conclusion

Although QSM has the potential to be a robust technology, care must be taken in assessing some smaller structures like the DN, the RN and the SN to avoid reconstruction bias based on slice thickness. We recommend using a slice thickness no greater than 2 mm to avoid a resolution-related reduction in susceptibility values. RII iron analysis showed a tighter age-related behavior than global iron analysis and appeared to be less susceptible to imaging parameters, field strength, or region drawing. For the first time, we showed that the local iron content in the GP increases with age (range 20–90 years) despite the global iron remaining roughly constant. Almost all structures showed a reduction in iron containing volumes with age except for the SN and DN. Finally, the results of this work show that a reasonable estimate for age-related iron behavior can be obtained from a large cross site, cross manufacturer set of data and can be used for correcting for age related iron changes when studying diseases like Parkinson’s disease and other iron related neurodegenerative diseases.

## Data Availability Statement

The raw data supporting the conclusions of this article will be made available by the authors, without undue reservation.

## Ethics Statement

The studies involving human participants were reviewed and approved by the First Affiliated Hospital of Dalian Medical University, Ruijin Hospital, Shanghai Jiao Tong University School of Medicine, and the First Affiliated Hospital of Zhengzhou University. The patients/participants provided their written informed consent to participate in this study.

## Author Contributions

YL: conceptualization, methodology, formal analysis, investigation, data curation, and writing – original draft. SS: conceptualization, software, validation, writing – original draft, and visualization. CZ, YM, and JC: investigation. KY and VP: methodology and software. SG: data curation and writing – review and editing. CW: writing – review and editing. NH: investigation and funding acquisition. FY: resources, supervision, project administration, and funding acquisition. EH: conceptualization, supervision, and writing – review and editing. All authors agreed to be accountable for the content of the work.

## Conflict of Interest

SS, SG, and EH were employed by Magnetic Resonance Innovations, Inc., and SpinTech, Inc. KY and VP were employed by MR Medical Imaging Innovations India. The remaining authors declare that the research was conducted in the absence of any commercial or financial relationships that could be construed as a potential conflict of interest.

## Abbreviations

QSM: quantitative susceptibility mapping
DGM: deep gray matter
GRE: gradient echo
HC: healthy controls
MS: multiple sclerosis
CNR: contrast-to-noise ratio
WM: white matter
FOV: field-of-view
SNR: signal to noise ratio
BET: brain extraction tool
3DSRNCP: 3D phase unwrapping algorithm
SHARP: sophisticated harmonic artifact reduction
TKD: truncated k-space division
CN: caudate nucleus
GP: globus pallidus
PUT: putamen
THA: thalamus
PT: pulvinar thalamus
RN: red nucleus
SN: substantia nigra
DN: dentate nucleus
FWHM: full-width half maximum
SPIN: Signal Processing in Nmr
ROI: region of interest
ANOVA: analysis of variance
GMD: gray matter density
GMV: gray matter volume
STAGE: STrategically Acquired Gradient Echo.
