# Supplementary material for: Iron Content in Deep Gray Matter as a Function of Age Using Quantitative Susceptibility Mapping: A Multicenter Study
Source: Front Neurosci. 2021 Jan 6;14:607705. doi: 10.3389/fnins.2020.607705 (PMC7815653; doi:10.3389/fnins.2020.607705)
Supplement: Supplementary file 1 [file Data_Sheet_1.pdf]

Supplementary Material

Table S1. The iron content of the PUT, RN, and SN from the k-space cropped lower resolution images were correlated with the iron content of the original images. The values of all the slopes and $\mathbf{R^2}$ are given in Table S1. The $\mathbf{R^2}$ values are all close to unity indicating a very good agreement between the measurements.

	**Right side**						**Left side**					
	**Site 1**		**Site 2**		**Site 3**		**Site 1**		**Site 2**		**Site 3**	
	**slope**	$\mathbf{R^2}$	**slope**	$\mathbf{R^2}$	**slope**	$\mathbf{R^2}$	**slope**	$\mathbf{R^2}$	**slope**	$\mathbf{R^2}$	**slope**	$\mathbf{R^2}$
**PUT**	1.01	0.99	0.95	0.99	0.98	0.99	0.99	0.99	0.94	0.99	0.99	0.99
**RN**	0.97	0.99	0.97	0.99	1	0.98	1	0.98	1.01	0.99	1.06	0.98
**SN**	0.97	0.99	0.93	0.99	1.02	0.98	0.99	0.98	0.99	0.99	1.02	0.98

PUT (Putamen), RN (Red Nucleus), and SN (Substantia Nigra).

Table S2. Mean susceptibility (ppb), standard deviation and standard error of the different DGM in subjects (age range 55-65 years) from 3 sites. The means in this age range are similar to each other for all three sites across all the structures. It should be noted that the age ranges for each site are different and narrow age ranges will lead to larger errors in the slope. When merged together into a single large data set, the slopes are much more accurately determined.

		**RII analysis**			**Global analysis**		
		**mean**	**std devn***	**std error**	**mean**	**std devn**	**std error**
**CN**	Site 1	95.99	7.79	1.25	46.16	10.92	1.75
	Site 2	98.93	10.02	0.72	45.74	12.87	0.92
	Site 3	99.23	8.32	1.25	44.27	9.66	1.46
**GP**	Site 1	236.76	24.91	4.04	134.18	23.98	3.84
	Site 2	229.52	21.6	1.69	115.46	24.13	1.77
	Site 3	240.98	23.47	3.67	128.93	21.16	3.25
**PUT**	Site 1	142.35	14.04	2.25	74.16	18.74	3.02
	Site 2	133.46	11.58	0.84	53.2	18.04	1.3
	Site 3	133.17	11.94	1.8	50.26	13.41	2.02
**THA**	Site 1	29.37	7.13	1.14	-0.06	6.89	1.1
	Site 2	27	7.91	0.57	-10.07	7.46	0.53
	Site 3	27.89	7.76	1.17	-8.57	6.64	1
**PT**	Site 1	81.01	6.99	1.15	43.67	14.62	2.34
	Site 2	82.9	7.96	0.71	33.04	18.61	1.33
	Site 3	81.65	8.11	1.42	36.68	16.13	2.44
**RN**	Site 1	175.47	13.86	2.57	90.2	30.97	4.96
	Site 2	175.23	12.31	0.93	110.47	32.97	2.35
	Site 3	176.92	15.88	2.45	123.42	33.61	5.07
**SN**	Site 1	197.29	14.71	2.42	105.84	30.21	4.84
	Site 2	204.28	15.42	1.12	130.08	30.28	2.17
	Site 3	206.2	16.99	2.56	134.85	28.05	4.25
**DN**	Site 1	148.96	13.48	3.09	48.53	29.62	5.08
	Site 2	156.97	15.74	1.15	102.54	28.2	2.04
	Site 3	153.72	11.88	1.81	100.33	24.02	3.62

CN (Caudate Nucleus), GP (Globus Pallidus), PUT (Putamen), THA (Thalamus), PT (Pulvinar Thalamus), RN (Red Nucleus), SN (Substantia Nigra), DN (Dentate Nucleus), and ppb (parts per billion) unit.

Table S3. Linear fitting equations for mean susceptibility (χ) (ppb) versus age for the global analysis.

	**χ=A×age+B**	**Error in A (ppb/year)**	**Error in B (ppb)**	***r***	***r*-CI**	***p*-value**
**CN**	$\chi=0.37\times age+22$	0.07	4.02	0.39	(0.33,0.46)	<0.001
**GP**	$\chi=-0.05\times age+124$	0.15	8.75	-0.03	(-0.11,0.05)	0.49
**PUT**	$\chi=0.51\times age+25$	0.11	6.29	0.35	(0.28,0.42)	<0.001
**THA**	$\chi=-0.42\times age+17$	0.04	2.56	-0.60	(-0.65,-0.55)	<0.001
**PT**	$\chi=-0.15\times age+44$	0.11	6.30	-0.11	(-0.19,-0.03)	<0.01
**RN**	$\chi=1.41\times age+23$	0.20	11.56	0.49	(0.43,0.55)	<0.001
**SN**	$\chi=0.92\times age+71$	0.19	10.91	0.37	(0.29,0.43)	<0.001
**DN**	$\chi=1.4\times age+9$	0.22	13.12	0.47	(0.4,0.53)	<0.001

r: Pearson correlation coefficient; *r*-CI: Confidence interval of r

Table S4. Linear fitting equations for global nuclei volume (V) (mm^3) vs age.

	**V=A×age+B**	**Error in A (mm^3/year)**	**Error in B (mm^3)**	***r***	***r*-CI**	***p*-value**
**CN**	V = -6.78×age+2279	2.16	125.93	-0.24	(-0.32,-0.17)	<0.001
**GP**	V = -7.66×age+2303	2.05	119.42	-0.29	(-0.36,-0.21)	<0.001
**PUT**	V = -18.8×age+4009	2.55	149.01	-0.51	(-0.56,-0.45)	<0.001
**THA**	V = -14.72×age+4423	3.77	220.42	-0.30	(-0.37,-0.22)	<0.001
**PT**	V = -6.89×age+963	0.88	51.16	-0.53	(-0.58,-0.47)	<0.001
**RN**	V = -1.08×age+247	0.25	14.36	-0.33	(-0.4,-0.26)	<0.001
**SN**	V = 2.14×age+367	0.64	37.39	0.26	(0.18,0.33)	<0.001
**DN**	V = 3.55×age+428	1.30	77.53	0.22	(0.14,0.3)	<0.001

r: Pearson correlation coefficient; *r*-CI: Confidence interval of r

Table S5. Linear fitting equations for volume (V) (mm^3) vs. age for RII Analysis.

	**V=A×age+B**	**Error in A (mm^3/year)**	**Error in B (mm^3)**	***r***	***r*-CI**	***p*-value**
**CN**	V=-0.48×age+438	1.31	76.51	-0.03	(-0.11,0.05)	0.47
**GP**	V=-2.64×age+390	1.17	67.98	-0.18	(-0.26,-0.1)	<0.001
**PUT**	V=-1.33×age+543	1.82	106.68	-0.06	(-0.14,0.02)	0.15
**THA**	V=-13.45×age+1997	2.46	143.80	-0.40	(-0.46,-0.33)	<0.001
**PT**	V=-0.39×age+108	0.66	37.38	-0.06	(-0.15,0.04)	0.24
**RN**	V=0.22×age+37	0.29	17.00	0.06	(-0.02,0.15)	0.14
**SN**	V=1.71×age+44	0.61	35.65	0.22	(0.15,0.3)	<0.001
**DN**	V=3.04×age+30	1.31	79.14	0.20	(0.11,0.28)	<0.001

r: Pearson correlation coefficient; *r*-CI: Confidence interval of r

Table S6. Linear fitting equations for total iron (I) (ppb × mm^3) versus age for the global analysis.

	**I=A×age+B**	**Error in A (in ppb×mm^3 /year)**	**Error in B (ppb)**	***r***	***r-CI***	**p-value**
**CN**	I=408.63×age+58384	162.41	9487.51	0.20	(0.12,0.27)	<0.001
**GP**	I=-890.54×age+277111	406.45	23742.05	-0.17	(-0.25,-0.09)	<0.001
**PUT**	I=531.96×age+123698	341.17	19928.81	0.12	(0.04,0.2)	<0.001
**THA**	I=-1400.84×age+58884	155.84	9102.44	-0.58	(-0.63,-0.53)	<0.001
**PT**	I=-330.82×age+39571	76.03	4436.17	-0.33	(-0.4,-0.26)	<0.001
**RN**	I=182.02×age+8691	44.86	2624.96	0.31	(0.23,0.38)	<0.001
**SN**	I=675.86×age+22358	147.96	8658.07	0.34	(0.27,0.41)	<0.001
**DN**	I=1075.62×age+-2236	225.04	13436.06	0.37	(0.3,0.44)	<0.001

r: Pearson correlation coefficient; *r*-CI: Confidence interval of r

Table S7. Linear fitting equations for total iron (I) (ppb × mm^3) versus age for the RII analysis.

	I=A×age+B	Error in A (ppb×mm^3/ year)	Error in B (ppb)	*r*	*r*-CI	*p*-value
**CN**	I=302.16×age+22850	138.77	8095.83	0.17	(0.09,0.25)	<0.001
**GP**	I=-430.18×age+78979	281.71	16389.47	-0.13	(-0.21,-0.04)	<0.001
**PUT**	I=375.42×age+38454	250.61	14609.73	0.12	(0.04,0.2)	<0.001
**THA**	I=-510.21×age+64247	107.79	6293.19	-0.36	(-0.42,-0.28)	<0.001
**PT**	I=-12.9×age+7265	52.65	2989.73	-0.02	(-0.12,0.07)	0.63
**RN**	I=79.89×age+4110	54.28	3215.36	0.13	(0.04,0.21)	<0.001
**SN**	I=420.72×age+5531	136.88	8025.78	0.24	(0.17,0.32)	<0.001
**DN**	I=561.39×age+679	229.88	13894.25	0.21	(0.12,0.29)	<0.001

r: Pearson correlation coefficient; *r*-CI: Confidence interval of r

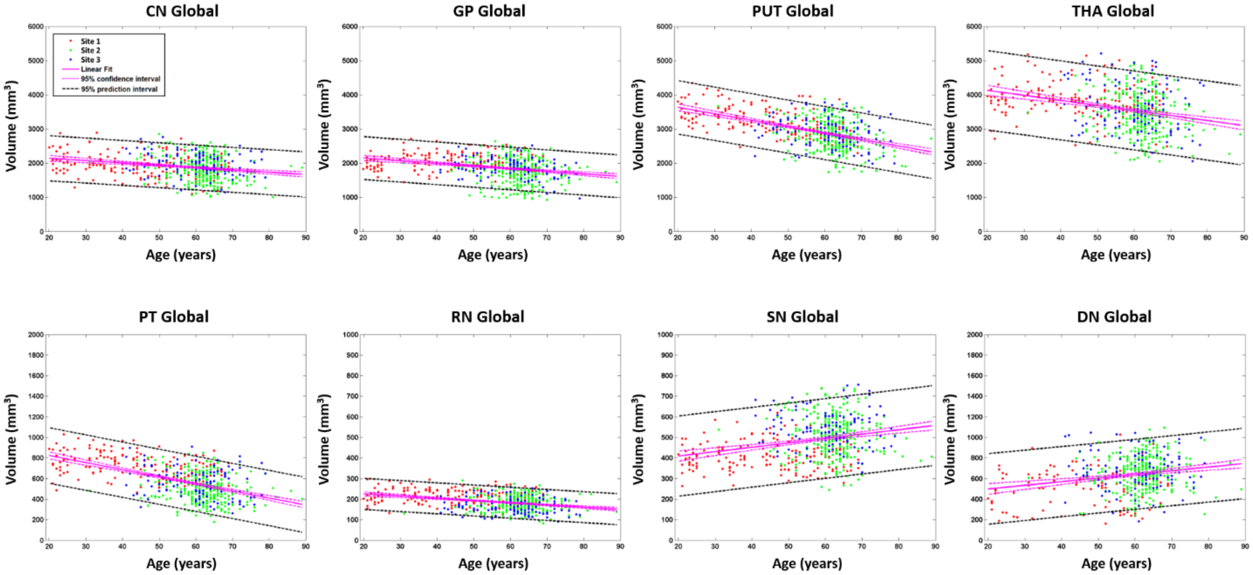

Figure S1. Volumes for the global analysis. The volumes (the average of the bilateral nuclei) and 95% confidence intervals and 95% prediction intervals are shown for each structure as a function of age. CN: caudate nucleus, GP: globus pallidus, PUT: putamen, THA: thalamus, PT: pulvinar thalamus, RN: red nucleus, SN: substantia nigra, DN: dentate nucleus.

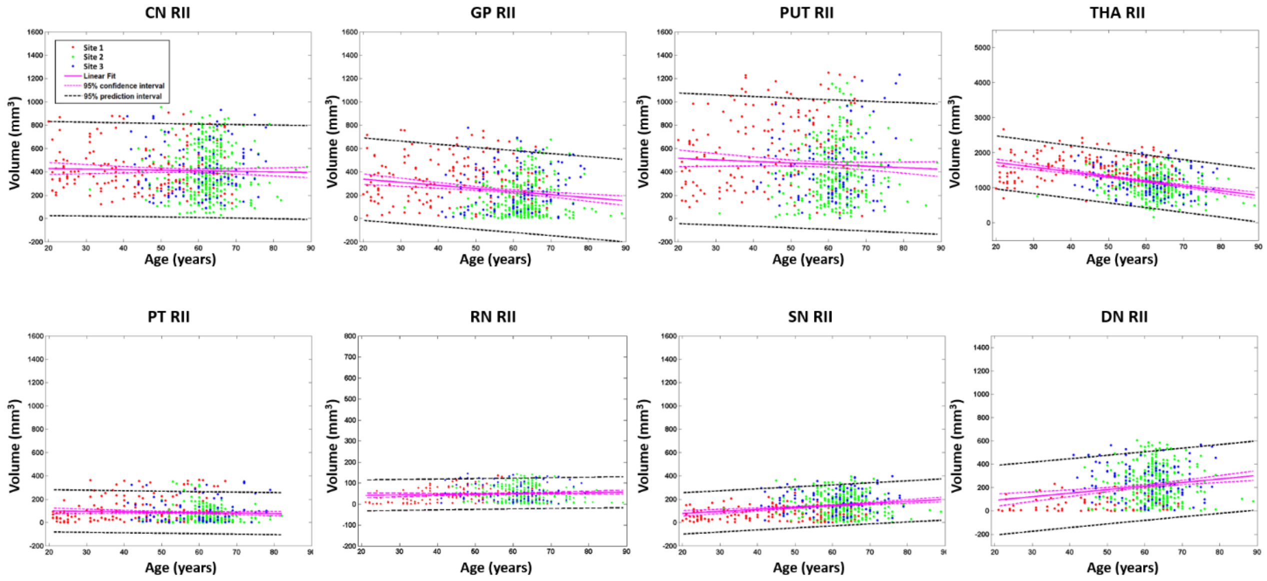

Figure S2. Volumes for the RII analysis. The volumes (the average of the bilateral nucleus) and 95% confidence intervals and 95% prediction intervals are shown for each structure as a function of age. CN: caudate nucleus, GP: globus pallidus, PUT: putamen, THA: thalamus, PT: pulvinar thalamus, RN: red nucleus, SN: substantia nigra, DN: dentate nucleus.

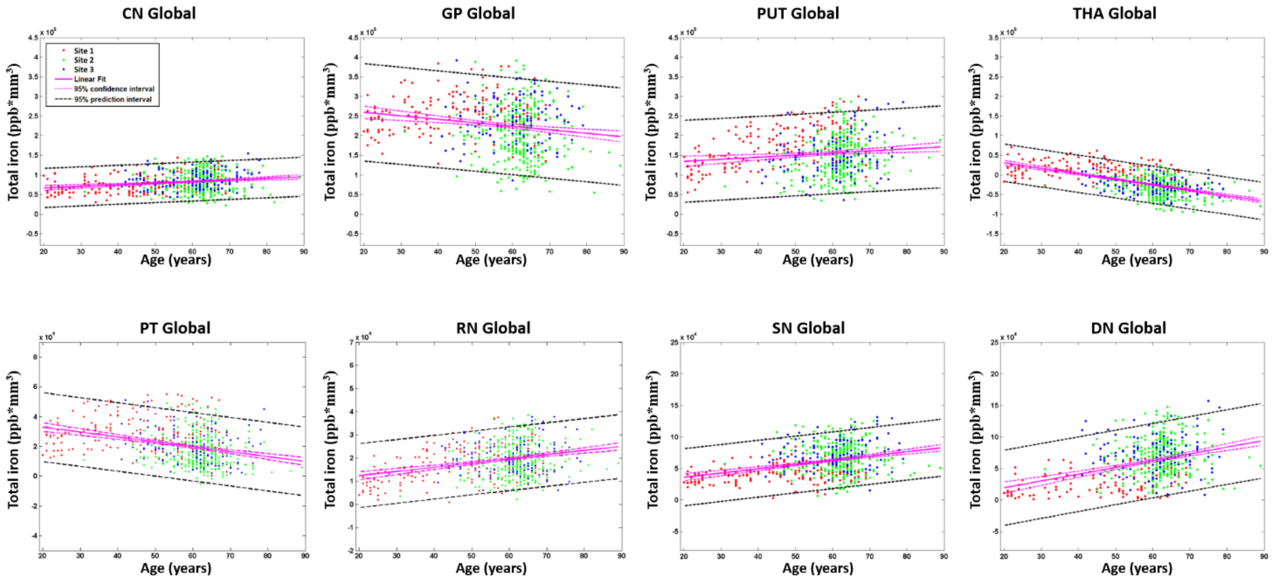

Figure S3. Total iron content (ppb×mm^3) from the global analysis. The iron and 95% confidence intervals and 95% prediction intervals are shown for each structure as a function of age. CN: caudate nucleus, GP: globus pallidus, PUT: putamen, THA: thalamus, PT: pulvinar thalamus, RN: red nucleus, SN: substantia nigra, DN: dentate nucleus.

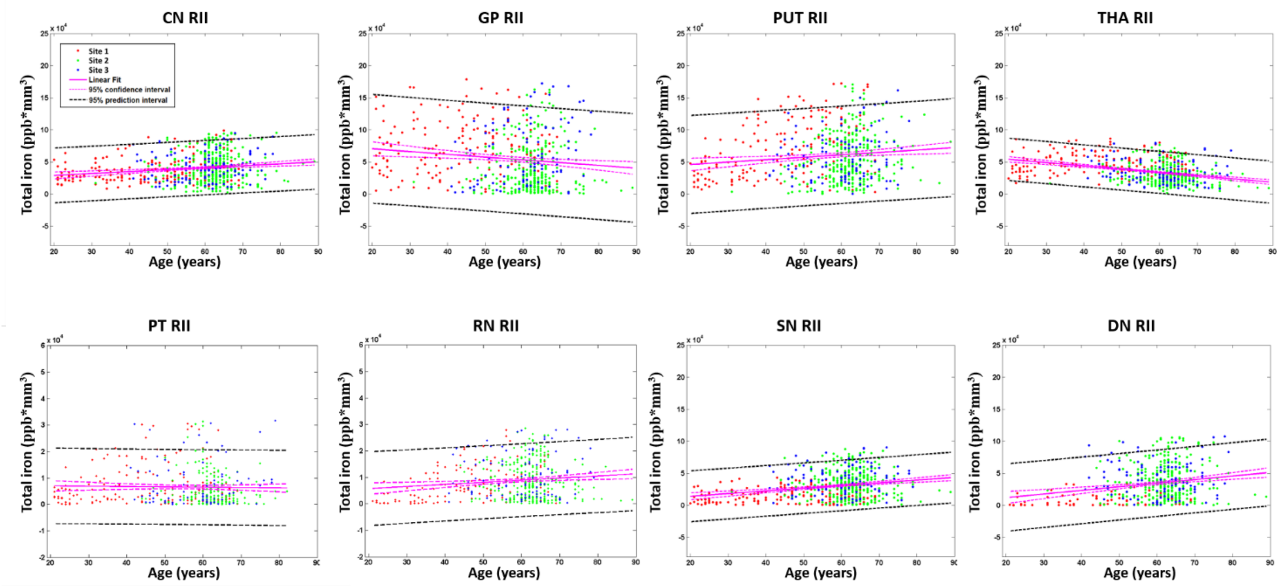

Figure S4. Total iron (ppb×mm^3) from the RII analysis. The iron and 95% confidence intervals and 95% prediction intervals are shown for each structure as a function of age. CN: caudate nucleus, GP: globus pallidus, PUT: putamen, THA: thalamus, PT: pulvinar thalamus, RN: red nucleus, SN: substantia nigra, DN: dentate nucleus.

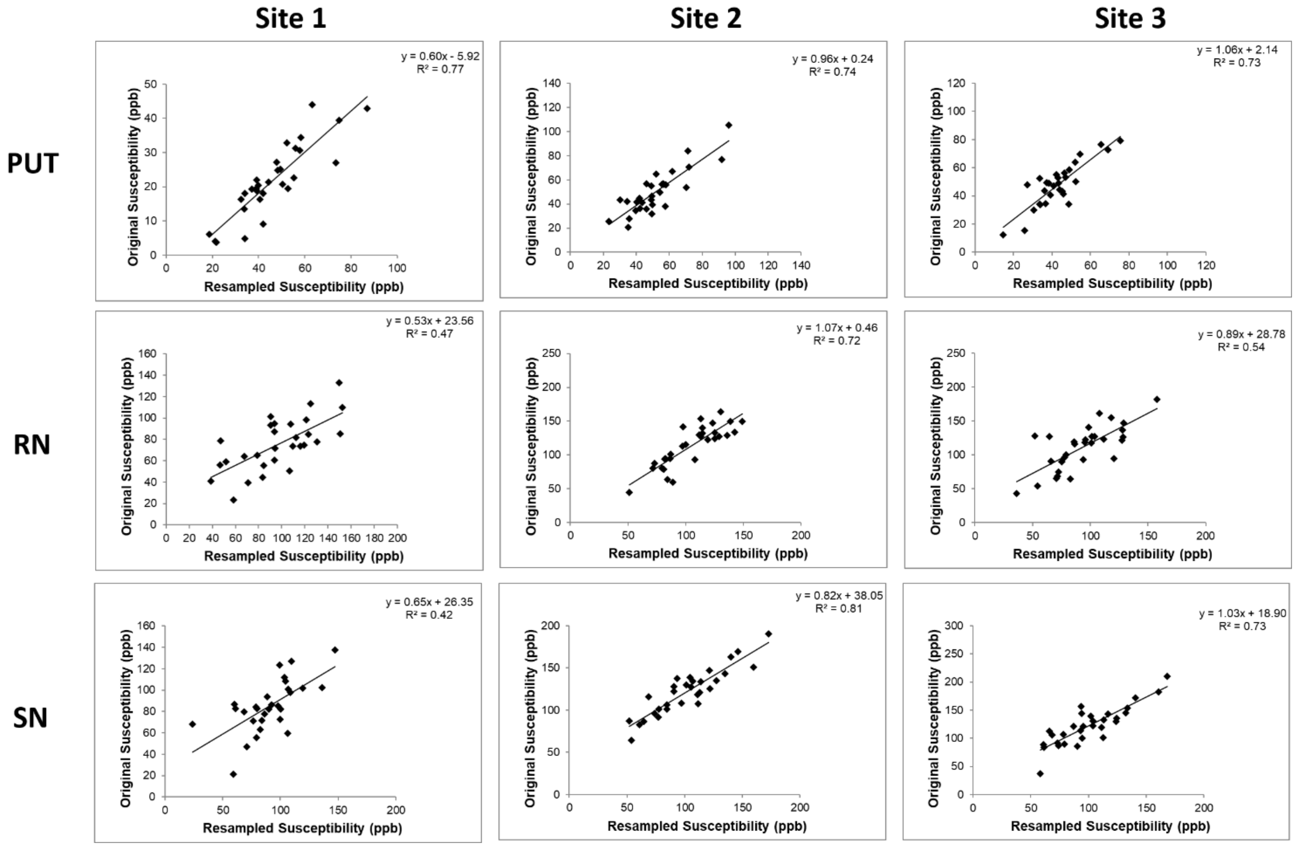

Figure S5. Comparison of the iron content of the PUT, RN, and SN between the k-space cropped lower resolution images and the original images. The ROI boundaries were redrawn in the resampled images but the partial volume effect was not taken into account.
